# Nobiletin Enhances Chemosensitivity to Adriamycin through Modulation of the Akt/GSK3β/β–Catenin/MYCN/MRP1 Signaling Pathway in A549 Human Non-Small-Cell Lung Cancer Cells

**DOI:** 10.3390/nu10121829

**Published:** 2018-11-26

**Authors:** Jeong Yong Moon, Le Van Manh Hung, Tatsuya Unno, Somi Kim Cho

**Affiliations:** 1 Subtropical/Tropical Organism Gene Bank, Jeju National University, Jeju 63243, Korea; owenmjy@jejunu.ac.kr (J.Y.M.); tatsu@jejunu.ac.kr (T.U.); 2 School of Biomaterial Science and Technology, College of Applied Life Sciences, Jeju National University, Jeju 63243, Korea; manhhung.levan@gmail.com3; 3 Faculty of Biotechnology, College of Applied Life Sciences, SARI, Jeju National University, Jeju 63243, Korea

**Keywords:** Adriamycin (ADR), A549 human non-small-cell lung cancer cells, multidrug resistance-associated protein 1 (MRP1), nobiletin (NBT)

## Abstract

Drug resistance is a major problem in the treatment of non-small-cell lung cancer (NSCLC). In this study, Kyoto Encyclopedia of Genes and Genomes (KEGG) pathway analysis was performed to identify the differentially expressed genes in Adriamycin (ADR)-resistant NSCLC A549/ADR cells compared with parental A549 cells. Among the tested phytochemicals, nobiletin (NBT) is able to overcome the ADR resistance of A549/ADR cells. NBT treatment decreased the expression of a neuroblastoma-derived MYC (MYCN) and multidrug resistance-associated protein 1 (MRP1) as well as downregulating Akt, GSK3β, and β-catenin. Consistent with these results, NBT treatment resulted in the accumulation of intracellular ADR. A combination index (CI) assay confirmed the synergistic effect of combined treatment with NBT and ADR in reducing the viability of A549/ADR cells (CI = 0.152). Combined treatment with NBT and ADR enhanced apoptosis in A549/ADR cells, as evidenced by increased caspase-3 activation, poly (ADP-ribose) polymerase (PARP) cleavage, and sub-G1 population compared to treatment with ADR alone. In vivo experiments using a mouse xenograft model revealed that combination therapy with NBT and ADR significantly reduced tumor volume by 84.15%. These data suggest that NBT can sensitize ADR-induced cytotoxicity against A549/ADR cells by inhibiting MRP1 expression, indicating that NBT could serve as an effective adjuvant agent for ADR-based chemotherapy in lung cancer.

## 1. Introduction

Lung cancer is one of the most serious cancers worldwide and is the most common malignancy in cancer-related deaths, leading to approximately 1.4 million deaths per year [1]. Lung cancer is considered to have the highest incidence and mortality with 1.8 million new cases and 1.6 million new deaths annually [2]. Lung cancers are classified into two major forms, small-cell lung cancer (SCLC) and non-small-cell lung cancer (NSCLC), based on microscopic appearance. NSCLC occurs more frequently and accounts for 85% of all cases of lung cancer [3]. Treatment strategies for lung cancer include radiotherapy, chemotherapy, and surgery [4]. The primary treatment approach for NSCLC is a surgical operation followed by chemotherapy to prevent recurrence [5]. However, in terms of the initial outcome, treatment for NSCLC remains inefficient compared to that for SCLC. Additionally, surviving NSCLC tumors subsequently display acquired drug resistance caused by multidrug resistance (MDR), i.e., the ability of tumor cells to develop resistance to various drugs [6]. Adriamycin (ADR) has been widely used as an anticancer drug for a large range of tumors, including lung cancer. Although SCLC is highly sensitive to ADR, NSCLC shows poor sensitivity to this chemotherapeutic agent [7,8]. Thus, it is vital to find alternative approaches to reduce the side effects caused by ADR and enhance its efficacy in clinical use.

Cancer cells expressing a high protein level of ATP binding cassette (ABC) transporters can attenuate the efficacy of treatment by actively pumping drugs outs of the cells, leading to the MDR phenotype [9]. Based on sequence homology and domain organization, these ABCs are subdivided into seven distinct subfamilies (ABCA–ABCG). Among these, multidrug resistance-associated protein 1 (MRP1), also known as ABCC1, was first identified from drug-resistant lung cancer cells that did not express ABCB1 (MDR1 or P-glycoprotein) [10]. MRP1 plays a role in drug resistance in various cancer including NSCLC tumors [11]. Regulation of the MRP1 gene at the 5′ untranslated promoter region is associated with various transcription factors, including neuroblastoma-derived MYC (MYCN) [12,13]. Control of MRP1 expression can be viewed as a potential way to improve sensitivity to chemotherapy. Indeed, ADR resistance in human bladder cancer cells by resveratrol has been reported to be partially associated with an alteration of MRP1 [14]. However, the regulation by phytochemicals of MRP1 and its underlying mechanism in drug-resistant cancer cells remains to be clarified.

Nobiletin (5,6,7,8,3′,4′-hexamethoxyflavone; NBT) is a major component of citrus fruits, particularly the peels of oranges (*Citrus sinensis*) [15]. NBT exhibits antiproliferative activities and suppresses invasion and migration of different cancer cell types, including human gastric adenocarcinoma, breast cancer, and lung cancer [16,17,18]. It has also been reported that NBT can sensitize the growth inhibition activities of the chemotherapeutic drug fluorouracil (5-FU) without affecting normal cells [16]. That study indicated that NBT may function by modulating and interacting with cellular targets that are associated with drug resistance. Consistent with these findings, more recently, NBT was shown to significantly sensitize ABCB1-overexpressing NSCLC cells to chemotherapeutic drugs by inhibiting the efflux function of ABCB1 [19]. However, the mechanisms underlying the ability of NBT to increase sensitivity to chemotherapeutic drugs remain unclear, and additional research on the functions and mechanisms of NBT as a chemosensitizer is needed.

In the present study, we used transcriptome analysis to identify the differentially expressed genes in A549/ADR cells compared with parental A549 cells, and then performed in vitro and xenograft animal studies to evaluate the ability of NBT to exhibit chemosensitizing activity against ADR. We found that NBT suppressed the Akt/GSK3β/β-catenin/MYCN signaling pathway and inhibited the expression of MRP1, leading to increased accumulation of ADR. These results are the first to demonstrate the underlying molecular mechanism by which NBT sensitizes ADR-induced cytotoxicity.

## 2. Materials and Methods 

### 2.1. Cell Culture 

Human non-small-cell lung cancer (NSCLC) A549 cells were generously provided by Professor Min-young Kim at the Faculty of Biotechnology, Jeju National University, Republic of Korea. Cells were cultured in Ham’s F-12K (Kaighn’s) Medium (F12K) supplemented with 10% heat-inactivated fetal bovine serum (FBS), 100 U/mL penicillin, and 100 μg/mL streptomycin at 37 °C in a humidified atmosphere under 5% CO_2_ in an incubator. The ADR-resistant cell line was established from the parental cell line by step-dose selection in vitro. A549 cells were treated with ADR at concentrations ranging from 0.03 to 0.5 μM over a period of 3 months. 

### 2.2. Cell Viability Assay 

Antiproliferative activity was determined by a cell viability assay. The effect of the samples on the viability of various cancer cell lines was determined by an 3-(4,5-Dimethylthiazol-2-yl)-2,5-Diphenyltetrazolium Bromide (MTT)-based assay. Exponential-phase cells were collected and transferred to a 96-well microtiter plate (5 × 10^4^ cells per mL) to detect cytotoxicity. The cells were incubated for 2 days with various concentrations of ADR with/without NBT. After incubation, 0.1 mg MTT (Sigma, St. Louis, MO, USA) was added to each well, and the cells were incubated at 37 °C for 4 h, after which the medium was carefully removed. Dimethyl sulfoxide (DMSO) (150 μL) was added to each well to dissolve the formazan crystals. After the crystals had dissolved completely, the plates were read at 570 nm using a Sunrise microplate reader (Tecan Group, Ltd., Salzburg, Austria). The percentage cell viability was calculated by the formula: mean value of (control group—treated group/control group) × 100%. All results were examined in triplicate for each concentration.

### 2.3. Transcriptome Analysis

Total RNA was extracted from A549 cells and A549/ADR cells using TRIzol reagent (Invitrogen, Carlsbad, CA, USA). RNA purity and concentration were checked using a UV1800 Spectrophotometer (SHIMADZU, Kyoto, Japan). Then, 1 μg of the total RNA was used to construct a library using the Illumina TruSeq mRNA Sample Prep Kit (Illumina, Inc., San Diego, CA, USA). Poly-T oligo-attached magnetic beads were added to purify the poly-A-containing mRNA molecules. RNA-Seq was performed by Macrogen, Inc. (Seoul, Korea) according to the manufacturer’s instructions. Prior to the transcriptome assembly, duplicated sequences were removed from the raw reads using FastUniq [20], and the human genome GRCh38 was indexed using Spliced Transcripts Alignment to a Reference (STAR) [21]. Trinity was used to assemble the reads into transcriptomes [22]. The abundance of each transcriptome was calculated using RNA-seq by Expectation Maximization (RSEM), which was used to determine significantly differentially expressed genes (DEGs) (*p* < 0.001 and at least a twofold change) using EdgeR; these were annotated with Trinotate (https://trinotate.github.io/) [23,24]. 

### 2.4. Functional Annotation of Differentially Expressed Genes (DEGs)

We analyzed Gene Ontology (GO) using the Database for Annotation, Visualization and Integrated Discovery (DAVID, http://david.abcc.ncifcrf.gov/) to investigate the primary function of the differential expression of messenger RNA (mRNAs) in A549/ADR cells. Furthermore, we also applied the Kyoto Encyclopedia of Genes and Genomes (KEGG) pathway analysis to classify DEGs into different functional pathways [25,26]. 

### 2.5. Analysis of the Effects of Drug Combinations 

The Chou–Talalay method was utilized to calculate the combination index (CI) using CalcuSyn software (Biosoft, Ferguson, MO, USA). CI values of <1, 1, and >1 indicate synergistic, additive, and antagonistic effects, respectively.

### 2.6. Intracellular Accumulation of ADR 

A laser scanning confocal microscope Olympus FV1200 (Olympus Coporation, Tokyo, Japan) was used to measure the intracellular accumulation of ADR. A549 or A549/ADR cells were cultured on a cover glass (ISO LAB 20 × 20 mm). After 24 h of incubation, the cells were treated with ADR (0.5 μM) alone or in combination with NBT (50 μM) and incubated for 6, 12, and 24 h. Subsequently, the culture medium was removed, and the cells were washed twice with phosphate-buffered saline (PBS). Cells were fixed in 4% formaldehyde for 20 min at room temperature and then washed twice with PBS. Nuclear DNA was stained with 10 μM Hoechst 33342. Imaging was carried out via fluorescence microscopy (Olympus Coporation, Tokyo, Japan) to compare the intracellular accumulation of ADR. For the flow cytometry analyses, ADR (0.5 μM) was added to A549 or A549/ADR cells and incubated with or without NBT (50 μM) for 6, 12, and 24 h. Cells were detached, re-suspended in 500 μL of PBS after washing in cold PBS, and analyzed by flow cytometry (BD FACS Aria, BD Biosciences, San Jose, CA, USA). MK571, a known MRP1 inhibitor, was used as a positive control.

### 2.7. Cell Cycle Analysis 

Cells (5 × 10^4^ cells/mL) were seeded 24 h before being treated with or without ADR for 48 h. After treatment, the cells were collected, fixed in 70% ethanol and kept at −20 °C. Before fluorescence-activated cell sorting (FACs) analysis, cells were washed in PBS (2 mM EDTA), resuspended in 0.5 mL PBS (2 mM EDTA) containing 1 mg/mL RNase and 50 mg/mL propidium iodide (PI), incubated in the dark for 30 min at 37 °C, and analyzed by FACScalibur flow cytometry (Becton Dickinson, Franklin Lakes, NJ, USA). Data from 10,000 cells were collected for each sample.

### 2.8. Western Blot Analysis 

Western blotting was performed as described previously [27]. Briefly, cell lysates were prepared in radioimmunoprecipitation assay (RIPA) lysis buffer. Most primary antibodies were used at 1:1000 dilution, except that β-actin (1:10,000) and anti-rabbit immunoglobulin G (IgG) secondary antibody (Vector Laboratories, Burlingame, CA, USA) were used at 1:5000 dilution. The membranes were analyzed using a BS ECL Plus kit (Biosesang Inc., Seongnam, Korea)

### 2.9. In Vivo Animal Studies 

Mice were maintained and used for experiments according to a protocol approved by the Institutional Animal Care and Use Committee of Jeju National University (Jeju, Korea). Then, 1  ×  10^6^ A549/ADR cells resuspended in a mixture of 100 µL Matrigel (Sigma-Aldrich, St. Louis, MO, USA) in PBS were subcutaneously inoculated of into the flanks of 6-week-old athymic BALB/c female nude mice (*n*  =  4/group). After the successful generation of tumor models, mice were treated with NBT (40 mg/kg), ADR (10 mg/kg) and their combination. The treatment was continued for up to 35 days. After that, the animals were sacrificed, and the tumors were removed from all animals and weighed.

### 2.10. Statistical Analysis 

Results are expressed as mean ± standard deviation (SD). One-way analysis of variance using SPSS v 12.0 software was applied. All assays were performed in triplicate. Values of *p* < 0.05 were considered statistically significant.

## 3. Results

### 3.1. Characteristics of A549/ADR Cells

To study the mechanism of ADR resistance in lung cancer, we first established an in vitro resistant cell line model by treating adenocarcinoma A549 with a gradually increasing concentration of ADR. The newly-established resistant cell line was designated as A549/ADR and evaluated for cytotoxicity against ADR by MTT assay.

**Figure 1:**
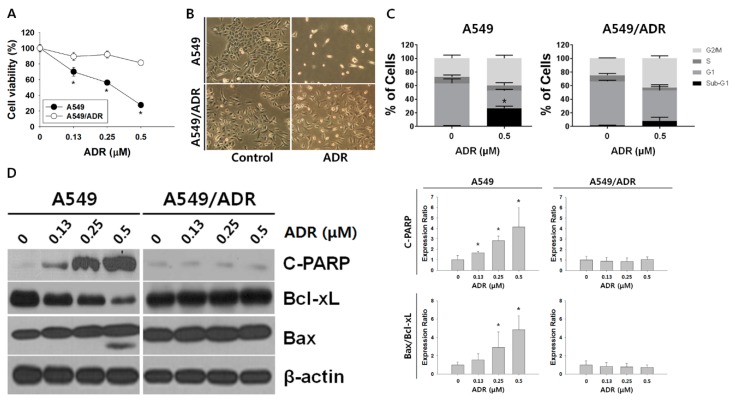
Comparison between A549 cells and ADR-resistant non-small cell lung cancer cells (A549/ADR). (**A**) MTT assay of A549 and A549/ADR cells treated with ADR (0.15–0.5 μM) for 48 h. (**B**) Morphological features of human lung A549 and A549/ADR cells treated with 0.5 µM ADR for 48 h. The cells were photographed at 200×. (**C**) A549 and A549/ADR cells were treated with ADR (0.5 μM) for 48 h and analyzed by flow cytometry after PI staining. The data shown are representative of three independent experiments indicating the quantification of sub-G1, G1, S, and G2/M populations. (**D**) Expression levels of apoptosis-related proteins were analyzed by Western blotting after 48 h of ADR treatment. β-actin was used as an internal control. The Western blotting intensities were quantified using ImageJ software. * *p* < 0.05. ADR: Adriamycin.

The mean IC_50_ value for ADR in the parent cell line A549 cells was 0.32 μM, whereas fewer than 80% of the A549/ADR cells with ADR treatment survived at concentrations up to 0.5 μM (Figure 1A). In addition, 0.5 μM ADR treatment induced not only dramatic decreases in cell numbers but also morphological changes in A549 cells, but not in A549/ADR cells (Figure 1B). We then examined the induction of apoptosis by ADR in both A540 and A549/ADR cells through cell cycle and Western blot analyses. The cell cycle control system is a major regulator of cell growth, and many cytotoxic agents are known to induce apoptosis through accumulation of the sub-G1 phase. In our study, 0.5 μM treatment of ADR resulted in a significant increase in the accumulation of sub-G1 in A549 compared to A549/ADR cells, at levels ranging from 1.02 ± 0.39% to 26.87 ± 3.03% and from 1.24 ± 0.69% to 8.26 ± 5.27%, respectively (Figure 1C). Western blot analysis demonstrated that the level of cleaved PARP was significantly increased and the expression of Bcl-xL was remarkably decreased (Figure 1D). These results indicate that ADR induced apoptosis by elevating the Bax/Bcl-xL ratio in the A549 cells, whereas the A549/ADR cell line showed tolerance to ADR.

### 3.2. Transcriptomic Analysis of ADR-Resistant Non-Small Cell Lung Cancer (A549/ADR) Cells

**Figure 2:**
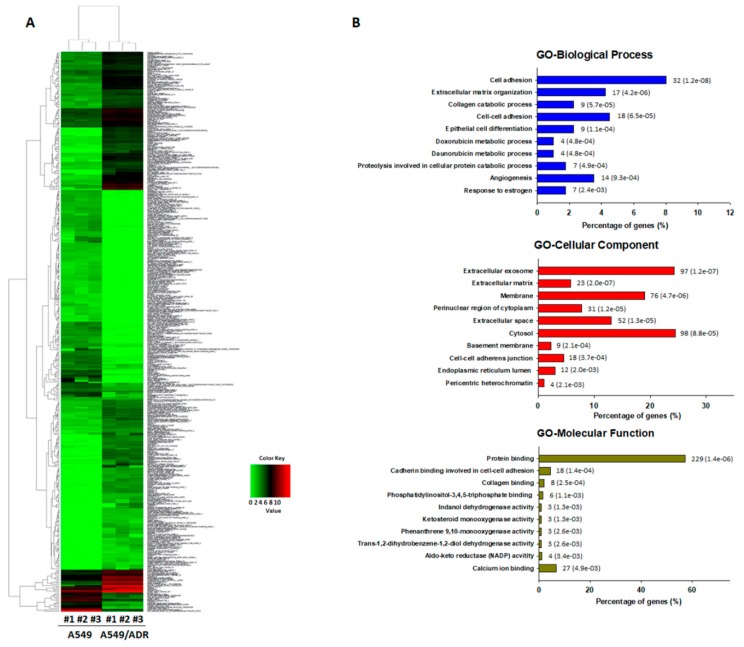
Analysis of differentially expressed genes (DEGs) in A549/ADR cells compared to A549 cells. (**A**) Heatmaps of gene expression data are shown for all samples. Representation of the log2-transformed values from 400 cellular genes identified as significantly altered in A549/ADR cells compared to A549 cells. (**B**) Top 10 pathways enriched by Gene Ontology (GO) analysis of DEGs in A549/ADR cells. The horizontal axis shows the percentage of annotated genes compared to the total gene number. The vertical axis shows the specific categories in each biological process, cellular component, and molecular function for GO as investigated by the Database for Annotation, Visualization and Integrated Discovery (DAVID) web tool and organized by *p*-values. The number shown on the right of each bar represents the number of genes, and the number in parentheses represents the *p*-value.

**Figure 3:**
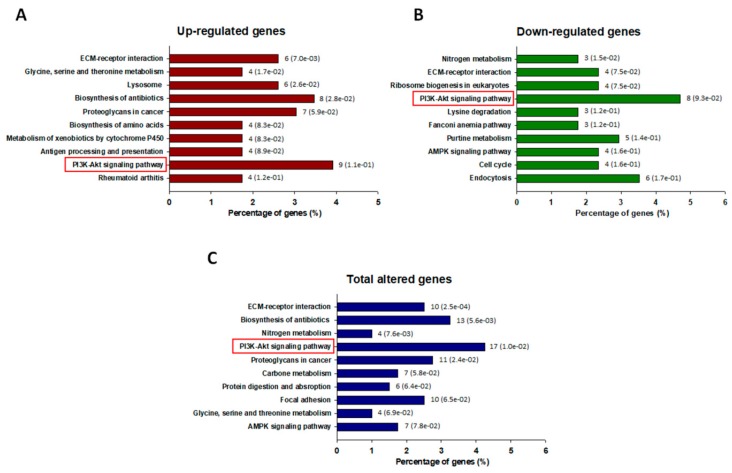
Top 10 altered pathways enriched in the Kyoto Encyclopedia of Genes and Genomes (KEGG) pathway analysis of A549/ADR cells. Pathways significantly enriched in (**A**) up-regulated genes, (**B**) down-regulated genes, and (**C**) total DEGs in A549/ADR cells vs. A549 cells analyzed in KEGG using DAVID. The horizontal axis shows the percentage of annotated genes compared to the total gene number. The vertical axis shows the pathways analyzed by KEGG analysis using DAVID. The pathways are organized by their *p*-values. The number shown on the right of each bar represents the number of genes, and the number in the parentheses represents the *p*-value. The red frame indicates the significant enrichment of the PI3K–Akt pathway from the KEGG pathway analysis.

We used the RNA-Seq method to further assess the transcriptome profile of ADR-resistant A549/ADR cells. Compared with parental A549 cells, 400 genes were differentially expressed in A549/ADR cells, including 170 down-regulated and 230 up-regulated genes (Figure 2A). After the DEGs were identified, the GO was analyzed using DAVID software to find the primary function of the differentially expressed mRNAs. Enrichment analysis showed that the GO categories including biological process (BP), molecular function (MF), and cellular component (CC) were most enriched in the categories related to “cell adhesion,” “extracellular exosome,” “membrane,” “cytosol,” and “protein binding” (Figure 2B). The KEGG is a database established to aid our understanding of the high-level functions and utilities of a biological system based on information at the molecular level, especially large-scale molecular datasets generated by genome sequencing and other high-throughput experimental technologies (http://www.genome.jp/kegg/) [28]. The KEGG database was used to determine the pathways regulated by ADR resistance in A549/ADR cells. An analysis of the up-regulated, down-regulated, and total DEGs identified PI3K–Akt as the most enriched pathway, with 9, 8, and 17 annotated genes, respectively (Figure 3A–C). 

### 3.3. Decrease in Intracellular Accumulation of ADR in A549/ADR Cells Compared to That in the Parental A549 Cells 

**Figure 4:**
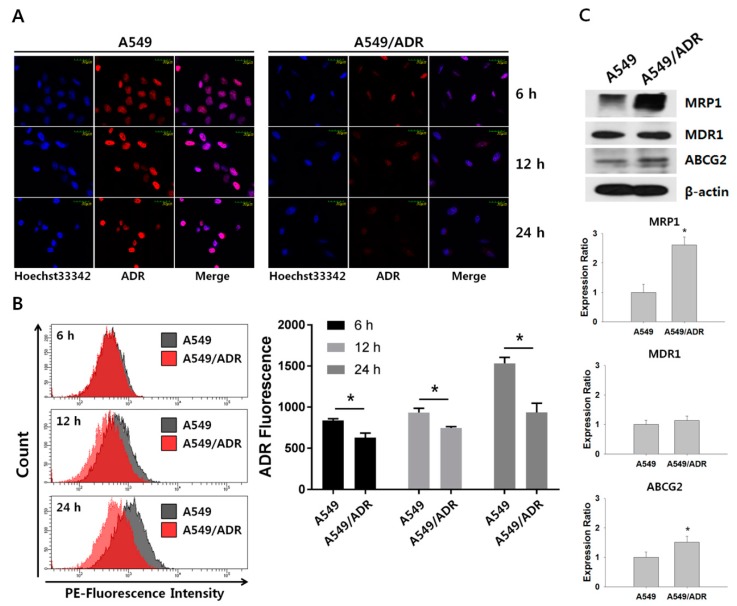
Decrease in intracellular ADR accumulation in A549/ADR cells. (**A**) Representative confocal images of A549 and A549/ADR cells treated with 0.5 μM ADR (red) for 24 h. (**B**) The histogram shows the ADR accumulation in A549 cells and A549/ADR cells measured by flow cytometry. (**C**) Expression levels of ABC transporters were analyzed by Western blotting analysis in A549 and A549/ADR cells. The Western blot intensities were quantified using ImageJ software. * *p* < 0.05.

To demonstrate how A549/ADR cells contribute to resistance to ADR-induced cytotoxicity, intracellular accumulation of ADR was examined under fluorescent microscopy. ADR is an auto-fluorescent compound that enables intracellular visualization. Figure 4A shows the fluorescence of intracellular accumulated ADR in A549 and A549/ADR cells after incubation with 0.5 μM of ADR for 6, 12, and 24 h. As presented in Figure 4B, intracellular fluorescence was similar between the two cell lines under the 6-h ADR treatment condition. However, increased ADR treatment time resulted in a marked increase in the intracellular fluorescence in A549 cells compared with A549/ADR cells. Because the expression levels of transporter proteins can affect the efflux and pharmacodynamics of major cell growth inhibitors that are applied in clinical oncology [29], the expression levels of MRP1, MDR1, and ABCG2 proteins were measured by Western blotting. As shown in Figure 4C, the expression levels of MRP1, MDR1, and ABCG2 increased by 2.61 ± 2.7, 1.13 ± 0.16, and 1.52 ± 0.20 fold, respectively, in A549/ADR cells, indicating that the decrease in the intracellular accumulation of ADR in A549/ADR cells was possibly due to the heightened expression of MRP1.

### 3.4. NBT Enhances Chemosensitivity of A549/ADR Cells to ADR and Suppresses the Akt/GSK3β/β-Catenin/MYCN/MRP1 Signaling Pathway 

**Figure 5:**
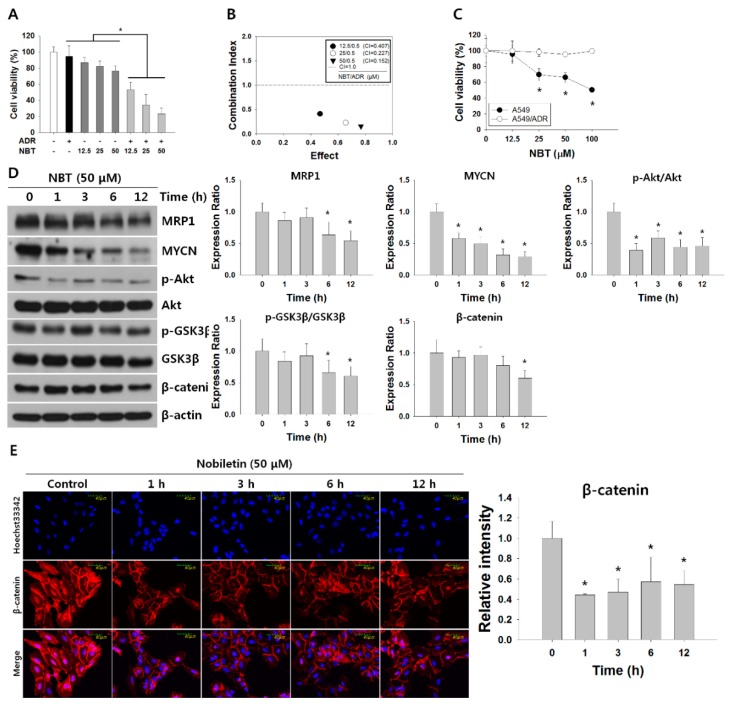
Nobiletin (NBT) enhances chemosensitivity of ADR by downregulating multidrug resistance-associated protein 1 (MRP1) expression through inhibiting Akt/GSK3β/β-catenin/ neuroblastoma-derived MYC (MYCN) in A549/ADR cells. (**A**) Cytotoxicity of ADR on A549/ADR cells treated with or without NBT as measured by MTT assay. (**B**) Combination index (CI) for various concentrations of NBT with ADR in A549/ADR cells. (**C**) Cytotoxicity of NBT in A549 and A549/ADR cells as measured by MTT assay. (**D**) MRP1 and Akt/GSK3β/β-catenin/MYCN signaling pathway expression levels in NBT-treated A549/ADR cells analyzed by Western blotting. (**E**) A549/ADR cells treated with NBT time dependently and analyzed by confocal immunofluorescence microscopy for Hoechst33342 (blue) and β-catenin (red) and their areas of overlap. Representative examples of three independent experiments are shown. Data are expressed as mean ± SD. * *p* < 0.05.

To overcome ADR resistance, we sought to identify the phytochemicals that exhibited chemosensitizing activities against ADR. Some phytochemicals were reported to have anticancer activity in lung cancer cells and xenograft models, and these substances that sensitize ADR anticancer activity were investigated [30,31,32,33,34,35,36]. The results suggested that a combined treatment of quercetin (QCT) or hesperidin (HPD) with ADR could not effectively inhibit cell proliferation; the inhibitory effects were similar to those for ADR monotherapy (Supplementary Figure S1A,B). However, as seen in Supplementary Figure S1C,D, the group of phytochemicals comprising NBT, d-limonene (LMN), and auraptene (ART) combined with ADR yielded inhibitory rate increases when compared to administration of ADR (0.5 μM) alone. In particular, combination treatment of NBT with ADR significantly decreased cell viability in A549/ADR cells in a concentration-dependent manner (Figure 5A). A CI assay confirmed the synergistic effect of combined treatment with NBT and ADR in reducing the viability of A549/ADR cells (Figure 5B). The findings showed a good synergistic effect of 50 μM NBT and 0.5 μM ADR (CI = 0.152). Next, we further investigated the underlying mechanism of the chemosensitizing activity of NBT against ADR in A549/ADR cells. Based on the observation that at 50 μM, NBT monotherapy did not exhibit an obvious cytotoxic effect on A549/ADR cells (Figure 5C), we hypothesized that NBT could improve the efficacy of ADR by increasing the accumulation of ADR in A549/ADR cells, and we investigated whether the expression level of MRP1 could be regulated by NBT. As shown in Figure 5D, after treatment with NBT, the expression levels of MRP1 and MYCN decreased in A549/ADR cells in a time-dependent manner, consistent with the previous report that MRP1 is a downstream transcriptional target of MYCN in neuroblastomas [12]. Moreover, the expression level and stabilization of MYCN protein are reported to be regulated through the Wnt/β-catenin and PI3K/Akt/GSK3β pathways. Akt attenuates GSK3β activity by phosphorylating GSK3β at Ser9, while active GSK-3β phosphorylates β-catenin to promote its ubiquitination and degradation in the β-catenin-dependent Wnt signaling pathway [37]. We investigated the phosphorylation of Akt and GSK3β and the changes in levels of β-catenin as well as its downstream target MYCN. Western blotting analysis showed a decrease in β-catenin and GSK3β phosphorylation, consistent with the downregulation of MYCN (Figure 5D). Additionally, there was a decrease in the level of nuclear accumulation of β-catenin, as detected by confocal microscopy with NBT treatment, when compared to the control (Figure 5E). These results suggest that NBT increases the intracellular accumulation of ADR, possibly by inhibiting the expression of MRP1 via suppression of the Akt/GSK3β/β–catenin/MYCN signaling pathway in A549/ADR cells.

### 3.5. NBT Enhances Intracellular Accumulation of ADR without Changing the Function of the Efflux Pump 

**Figure 6:**
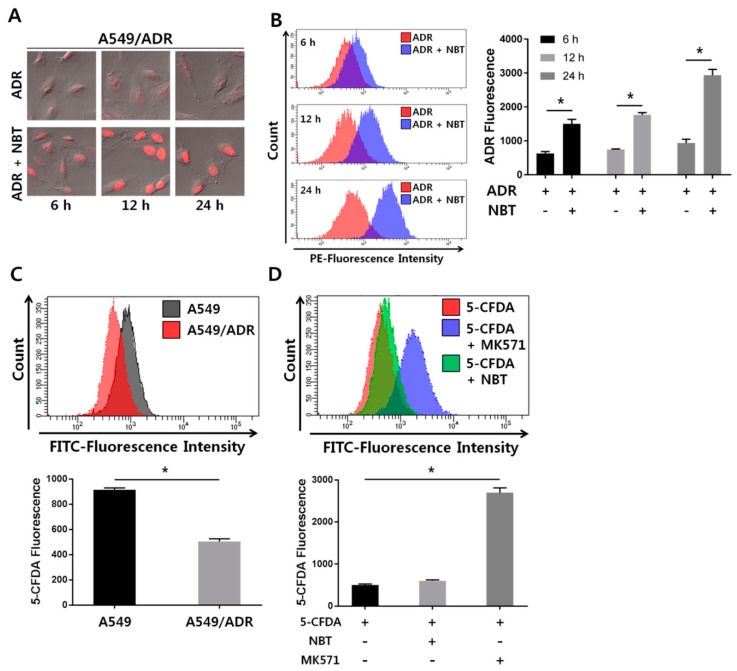
NBT enhances intracellular accumulation of ADR in A549/ADR cells. (**A**) Confocal microscopy images showing ADR accumulation in A549/ADR cells treated with 0.5 μM ADR alone (red) or ADR in combination with 50 μM NBT for 6, 12, and 24 h. (**B**) The histogram shows the relative fluorescence intensity of ADR uptake in A549/ADR cells treated with/without 0.5 μM ADR. (**C**) Intracellular 5-carboxyfluorescein (5-CF) retention as a measure of MRP1 activity in A549 and A549/ADR cells. (**D**) A549/ADR cells were treated with 5-carboxyfluorescein diacetate (5-CFDA) (10 μM) for 30 min with or without the addition of NBT (50 μM), MK571. * *p* < 0.05.

**Figure 7:**
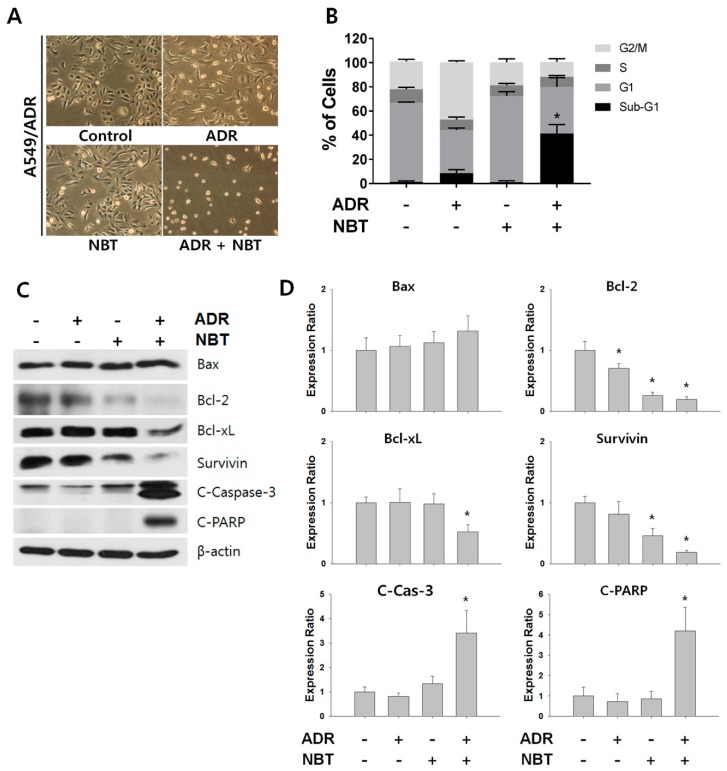
NBT enhances the apoptosis-initiating potential of ADR in A549/ADR cells. (**A**) Morphological features of A549/ADR cells after treatment with ADR (0.5 μM), NBT (50 μM), or both. (**B**) A549/ADR cells treated with ADR alone (0.5 μM), NBT alone (50 μM), and a combination of both were subjected to cell cycle analysis by flow cytometry using propidium iodide (PI) staining. (**C**) Expression levels of proteins were analyzed by Western blotting. β-actin was used as an internal control. (**D**) The intensities of the Western blotting bands were quantified using ImageJ software. * *p* < 0.05.

Next, we examined the effect of NBT on the intracellular accumulation of ADR by fluorescence microscopy. As shown in Figure 6A, intracellular levels of ADR were low in the A549/ADR cell line in the absence of NBT. Nonetheless, treatment with 50 μM NBT significantly increased the intracellular accumulation of ADR in a time-dependent manner. Furthermore, ADR uptake analysis by flow cytometry indicated that A549/ADR cells accumulated increased amounts of ADR in the presence of NBT, as seen by increased fluorescence intensity in the combination treatment group relative to the group treated with ADR alone (Figure 6B). Based on these results, we suggest that increased intracellular accumulation of ADR by treatment with NBT may serve as a mechanism to overcome ADR resistance in A549/ADR cells, and we hypothesize that this phenomenon may occur due to modulation of MRP1 activity or regulation of gene or protein expression. The activity of MRP1 involves the efflux of chemotherapeutic drugs from tumor cells into the surrounding environment [38]. As shown in Figure 7, the chemosensitizing activity of NBT can be achieved by antagonizing the function of the efflux pump or by decreasing the expression level of MRP1. Therefore, we sought to determine the activity of MRP1 in the presence or absence of NBT. It is known that 5-carboxyfluorescein diacetate (5-CFDA) is a well-established MRP1-specific substrate. CFDA diffuses into cells where it is cleaved by intracellular esterases, resulting in fluorescent 5-carboxyfluorescein (5-CF), which can be determined by flow cytometry [39]. To better clarify the abovementioned MRP1 effects, we sought to test the intracellular accumulation of 5-CF in A549 and A549/ADR cells. As shown in Figure 6C, the flow cytometry results indicated greater 5-CF accumulation in A549 cells. However, 5-CF accumulation was only weakly increased in A549/ADR cells despite treatment with NBT, whereas MK571, an MRP1-specific inhibitor, increased the accumulation of 5-CF (Figure 6D). These results indicate that the main outcome of NBT is not an increase in the intracellular accumulation of ADR through inhibition of MRP1 activity in A549/ADR cells. Thus, the sensitizing activity of ADR-induced cytotoxicity by NBT was likely not caused by inhibition of the drug efflux function of MRP1.

### 3.6. Combination Therapy with NBT and ADR Induced Apoptosis in A549/ADR Cells and Significantly Reduced Tumor Volume of A549/ADR Cells in Nude Mice 

**Figure 8:**
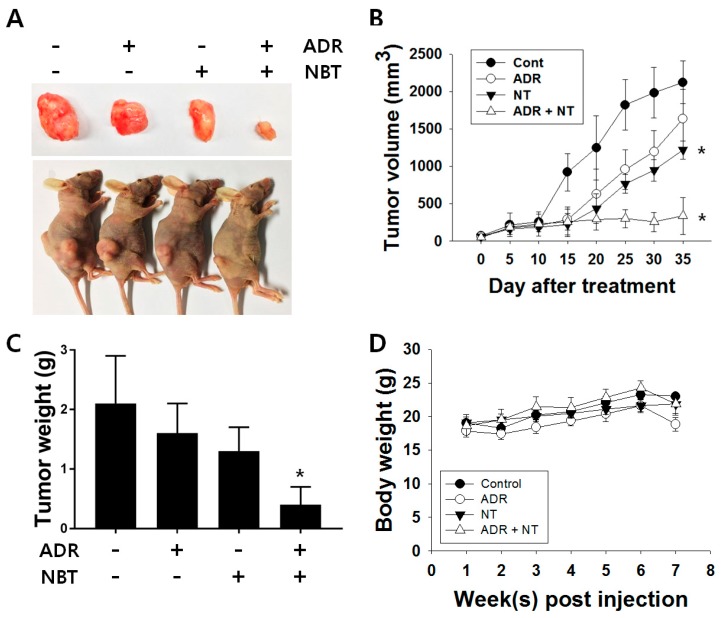
Antitumor effect of NBT in a nude mice xenograft tumor model. (**A**) Representative images of the tumor burden obtained from each group. (**B**) The tumor volume in each group was assessed by calipers and calculated as the length × width × width × 0.5. (**C**) Each bar represents the mean ± standard error of mean (SEM) of the tumor weight of four groups. (**D**) There were no significant changes in the body weights of the mice during the ADR and NBT treatment periods. * *p* < 0.05.

There was no significant change in cell morphology in either cell line treated with NBT alone or with ADR monotherapy, whereas when the cells treated with the combination therapy of NBT and ADR underwent morphological changes similar to apoptosis (Figure 7A). NBT enhanced ADR-induced apoptosis through an increase in the sub-G1 phase in A549/ADR cells (Figure 7B). Western blotting analysis showed that the combined treatment with NBT and ADR was more effective than that with either agent alone in inducing the cleavage of caspase-3 (3.41 fold) and c-PARP (4.20 fold) and the decrease in MRP1 (0.54 fold), survival (0.66 fold), and Bcl-xL (0.52 fold) in A549/ADR cells (Figure 7C). Additionally, to evaluate the effect of NBT on tumor growth, athymic nude mice inoculated with A549/ADR cells were treated with ADR (10 mg/kg) alone or in combination with NBT (40 mg/kg). Treatment of nude mice with ADR alone did not significantly inhibit tumor growth compared to the vehicle-treated control group. The tumor volume with ADR alone was 22.93 ± 18.42% lower than that of the vehicle-treated control group, and the tumor volume was reduced by 42.61 ± 5.73% in the NBT alone treatment group. On the other hand, the combined administration of ADR and NBT significantly reduced the tumor volume by 84.15 ± 11.54% compared to the vehicle-treated control group (Figure 8A,B). Consistent with the decrease in tumor volume, tumor weight was significantly reduced in the ADR and NBT combination treatment groups compared to those receiving ADR or NBT alone and vehicle-treated controls (Figure 8C). In addition, the body weight was not decreased in all four mouse groups, indicating the absence of any obvious systemic toxicity (Figure 8D).

## 4. Discussion

Although chemotherapy agents have been used successfully in a variety of cancer treatments, chemotherapy resistance is a major obstacle to effective cancer treatment. ADR (or doxorubicin) is the most widely used anticancer drug for a wide range of tumors, including lung cancer, and resistance to ADR is a prime example of anticancer drug resistance. While ADR shows high activity against SCLC, it shows relatively limited efficacy with NSCLC, which accounts for 85% of all lung cancer patients [8]. One cause of this low efficacy is acquired MDR. The overexpression of membrane transport proteins that effectively remove the chemotherapeutic drugs contributes to the mechanisms by which tumor cells acquire drug resistance. Among the most commonly known MDR-related membrane transporters are the ABC transporter superfamily, which includes P-gp (MDR-1) and multidrug resistant-associated protein (MRP-1). MRP-1, which was originally isolated from a doxorubicin-selected lung cancer cell line, mediates resistance to a broad range of anticancer drugs. As MRP-1 gene expression increases in various cancers including NSCLC, a combination of anti-cancer agents with MRP inhibitors to limit drug efflux is an obvious approach for the development of alternative chemotherapy treatments. Besides being inefficient, ADR also causes congestive heart failure when used at high doses, a major adverse effect [40]. Therefore, there is a need for novel therapeutic strategies that can minimize the dose and reduce the cytotoxicity of doxorubicin, and enhance its therapeutic efficacy against NSCLC cells.

After the establishment of ADR-resistant A549/ADR cells by exposing A549 adenocarcinoma cells to increasing doses of ADR, we compared the ADR toxicity profiles of A549 cells and A549/ADR cells by an MTT assay and cell cycle and Western blot analyses (Figure 1). A549/ADR cells showed higher cell viability, a reduced sub-G1 population, lower expression of pro-apoptotic (c-PARP, Bax) and higher expression of anti-apoptotic proteins (Bcl-xL). Among three commonly-upregulated ABC transporter genes in resistant cancer cell lines, we identified significant overexpression of MRP1 in A549/ADR cells (Figure 4C). These results suggest that overexpression of MRP1 protein may underlie A549/ADR cells’ resistance to ADR and the concomitant anti-apoptosis. Thus, based on the assumption that modulation of MRP1 protein expression is crucial in overcoming drug resistance to ADR, we performed transcriptome analyses of RNA sequencing data to compare overall gene expression patterns between the two cell lines. The major differences between the two cell lines as analyzed by the GO database confirmed the drug-resistant characteristic of A549/ADR cells and further provided evidence of the unique characteristics of A549/ADR cells compared to A549 cells in terms of changes in gene expression (Figure 2B). Previously, Fang et al. reported transcriptome analysis of cisplatin-resistant A549 in comparison with its parental cell line, demonstrating that the PI3K–Akt pathways, the mitogen-activated protein kinase (MAPK) pathway, and cell invasion pathways were enriched, and the highest number of DEGs were found in cisplatin-resistant A549 [41]. In accordance with the cisplatin-resistant A549, cisplatin-resistant hepatocellular carcinoma HepG2 was also most enriched in PI3K–Akt and cancer pathways based on the KEGG transcriptome analysis [41]. Interestingly, our KEGG pathway analysis also showed that the potentially oncogenic PI3K-Akt signaling pathway was significantly upregulated in the A549/ADR cell line compared to the A549 cell line (Figure 2B). These results further supported the importance of the PI3K-Akt pathway in controlling resistance against various types of chemotherapeutic agents, including cisplatin and ADR, in various cancer types [42,43,44]. However, in addition to the PI3K/Akt pathway and ABC transporters, we also witnessed changes in several proven multidrug resistance-associated pathways, including the extracellular matrix (ECM)-receptor interaction in A549/ADR cells and the AMP-activated protein kinase (AMPK) signaling pathway (Figure 3C). 

As shown in Figure 4, expression of MRP1 was greatly increased in association with decreased intracellular accumulation of ADR in A549/ADR cells. Based on these results and the results of the transcriptome analysis, we focused on the role of NBT in the regulation of the PI3K–Akt pathway and the ABC transporter (MRP1) to clarify the synergistic effects with ADR in A549/ADR cells. Interestingly, our results showed that the amount of MRP1 expression increased selectively among the ABC transporters, including MDR1 and ABCG2 (Figure 4C). As modulation of the expression of the ABC transporter is quite complex, selective downregulation of this transporter could represent a promising approach to novel chemotherapies. In particular, MRP1 is well known to play a crucial role in multidrug resistance and is over-expressed in a variety of cancers. It has been reported that the expression of MRP1 is highly correlated with the expression of MYCN and that MRP1 can be regulated by MYCN at the transcription level [12]. Interestingly, in our study, the level of MYCN was significantly higher in A549/ADR cells as compared to A549, and the expression of MYCN was down-regulated within 4 h following NBT treatment in A549/ADR cells (Supplementary Figure S2). This allowed us to speculate that MYCN is an important regulator of MRP1 expression in A549/ADR cells. 

Several researchers have reported on the role of MYCN in the progression of other tumors including neuroblastoma, and the development of new therapies targeting MYCN could be very attractive [45,46]. However, MYCN has not attracted much attention in the treatment of lung cancer. Recently, Liu et al. found that up-regulation of MYCN expression was associated with a poor clinical outcome in NSCLC patients [47]. Binding of the ubiquitin ligases to the *N*-terminal conserved phosphodegron domain (CPD) is required for initiation of proteasomal degradation of MYC-family proteins. In oncogenesis, the stabilization of MYC-family proteins can be controlled by phosphorylation within this region at threonine 58 (T58) and serine 62 (S62) by GSK3β and MAPK, respectively [48,49,50]. Phosphorylation at S62 serves as priming for GSK3β, which subsequently phosphorylates T58 to initiate Fbxw7-mediated degradation [51]. GSK3β is in turn inhibited via phosphorylation by Akt. As a result, signaling via PI3-kinase and Akt stabilizes MYCN and protects it from proteasomal degradation [45,52]. In addition to the translational regulation of MYCN by Akt–GSK3β signaling, the level of MYCN also can be regulated by GSK3β at the transcriptional level via a well-known modulator of β-catenin [53]. Chromatin immunoprecipitation assays showed that β-catenin was associated with conserved DNA binding sites for T-cell factor/lymphoid enhancer-binding factor (TCF/LEF) proteins, which are located in the MYCN promoter, indicating direct regulation of the MYCN promoter by canonical Wnt signaling [54,55]. Consistent with these previous studies, in our study, NBT induced down-regulation of MYCN either by Akt/GSK3β or by the Akt/GSK3β/β–catenin pathway axis (Figure 8). However, further studies are required to confirm that the regulation of the MRP1 gene expression is directly associated with the MYCN transcription factor at its promoter region and to investigate how NBT plays an important role in this regulation.

Above all, in this study, in vivo experiments using a xenotransplant model showed that the combined treatment significantly decreased the tumor volume and weight, which is consistent with the in vitro results. Moreover, the weight of the mice showed that the combination treatment did not cause significant systemic toxicity to the mice. In conclusion, our results suggested that NBT can act as an effective chemosensitizer against ADR in the A549/ADR cell line.

## Supplementary Materials

The following are available online at http://www.mdpi.com/2072-6643/10/12/1829/s1, Figure S1: Effect of the combined treatment with phytochemicals and ADR on cell proliferation in A549/ADR cells. Cytotoxicity of ADR treated with or without phytochemicals (A: Quercetin; QCT. B: Hesperidin; HPD. C: d-Limonene; d-LMN. D: Auraptene; ART) as measured by MTT assay. Representative examples of three independent experiments are shown. Data are expressed as mean ± SD. Figure S2: MYCN is highly expressed in A549/ADR cells and is down-regulated following NBT treatment. (A) The basal expression levels of MYCN and MRP1 in A549 and A549/ADR cells were shown by Western blotting analysis. (B) Western blotting analysis of MYCN levels after NBT treatment for 1, 2, and 3 h.
