# Supplementary material for: Nobiletin Enhances Chemosensitivity to Adriamycin through Modulation of the Akt/GSK3β/β–Catenin/MYCN/MRP1 Signaling Pathway in A549 Human Non-Small-Cell Lung Cancer Cells
Source: Nutrients. 2018 Nov 26;10(12):1829. doi: 10.3390/nu10121829 (PMC6316077; doi:10.3390/nu10121829)
Supplement: Supplementary file 1 [file nutrients-10-01829-s001.pdf]

Supplementary Materials: Nobiletin Enhances A549 Cell Chemosensitivity to Adriamycin through Modulation of the Akt/GSK3β/β–Catenin/MYCN/MRP1 Signaling Pathway in Human Non-Small-Cell Lung Cancer

Jeong Yong Moon, Le Van Manh Hung, Tatsuya Unno and Somi Kim Cho

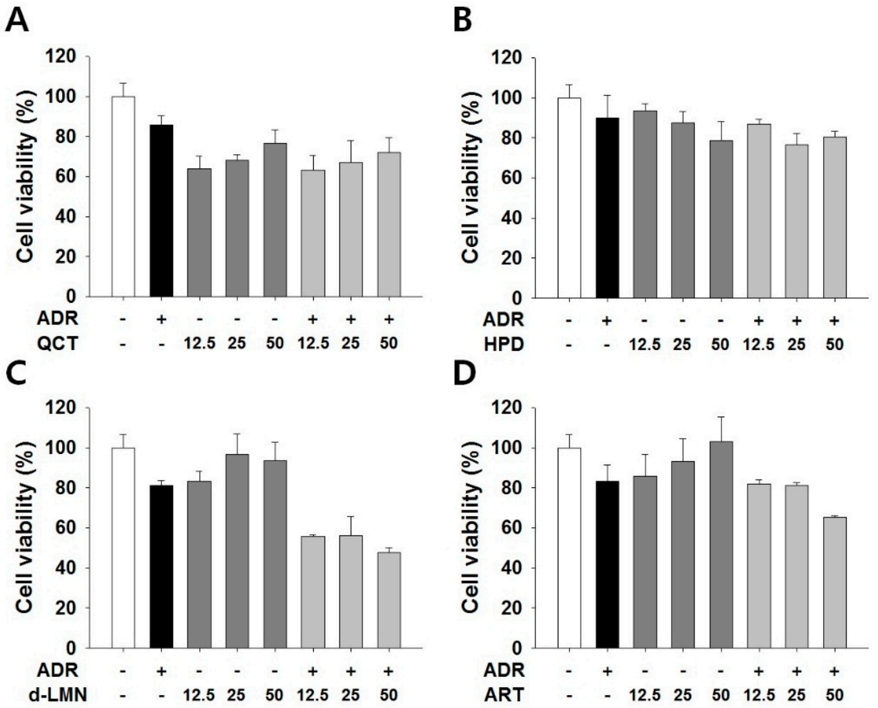

Figure S1. Effect of the combined treatment with phytochemicals and ADR on cell proliferation in A549/ADR cells. Cytotoxicity of ADR treated with or without phytochemicals (**A**) Quercetin; QCT. (**B**) Hesperidin; HPD. (**C**) d-Limonene; d-LMN. (**D**) Auraptene; ART as measured by MTT assay. Representative examples of three independent experiments are shown. Data are expressed as mean ± SD.

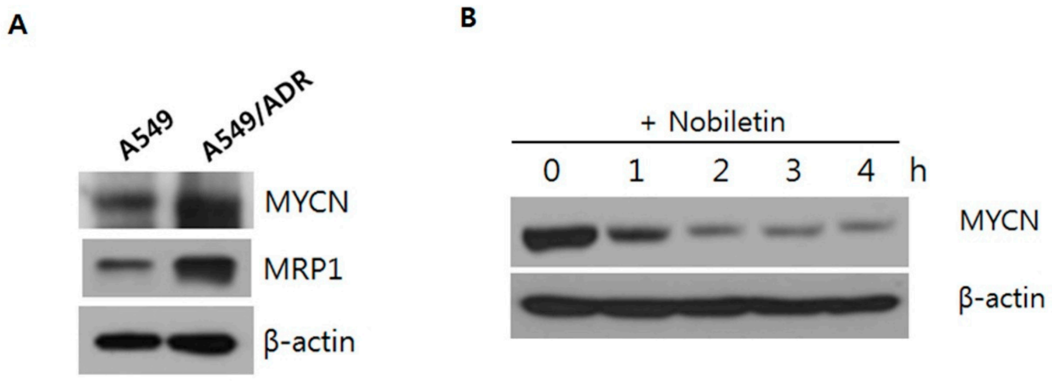

Figure S2. MYCN is highly expressed in A549/ADR cells and is down-regulated following NBT treatment. (**A**) The basal expression levels of MYCN and MRP1 in A549 and A549/ADR cells were shown by Western blotting analysis. (**B**) Western blotting analysis of MYCN levels after NBT treatment for 1, 2, and 3 h.
